# Using the Sustainable Development Capacity of Key Counties to Guide Rural Revitalization in China

**DOI:** 10.3390/ijerph20054076

**Published:** 2023-02-24

**Authors:** Jingru Chen, Hengyuan Zeng, Qiang Gao

**Affiliations:** 1 College of Economics and Management, Nanjing Forestry University, Nanjing 210037, China; 2 School of Economics, University of Bristol, Bristol BS8 1TU, UK

**Keywords:** rural revitalization, key counties to receive assistance, county sustainable development capacity (CSDC), restriction recognition

## Abstract

Counties are the basic unit for addressing unbalanced development in a region, revitalizing rural areas, and promoting the integrated development of urban and rural areas. Despite the importance of county-level research, few studies have been conducted research at such a small scale. To address this gap in knowledge, this study constructs an evaluation system to measure the county sustainable development capacity (CSDC) of counties in China, identify constraints to development, and provide policy suggestions to promote the counties’ long-term stable development. Specifically, the CSDC indicator system was based on the regional theory of sustainable development and included economic aggregation capacity, social development capacity, and environmental carrying capacity. This framework was applied to 103 key counties to receive assistance in pursuing rural revitalization in 10 provinces in western China. The AHP–Entropy Weighting Method and TOPSIS model were employed to inform the scores of CSDC and its secondary indicators, and ArcGIS 10.8 was used to portray the spatial distribution of CSDC and to classify these key counties into categories that guided specific policy recommendations. The results demonstrate a high degree of unbalanced and inadequate development in these counties and that rural revitalization efforts can be targeted to increase the speed of development. It is crucial to follow the recommendations that conclude this paper to promote sustainable development in areas that have been lifted out of poverty and to revive rural areas.

## 1. Introduction

The United Nations 2030 Agenda for Sustainable Development, adopted in 2015, established 17 Sustainable Development Goals (SDGs). Article 1 of this document proposes “No Poverty” and Article 10 “Reduced Inequalities” [1]. In China, rural revitalization is a national strategy proposed after the middle and late stages of China’s modernization. It is a social project with rural areas as the basic area, farmers as the main population, and agriculture as the main industry, with the core goal of modernizing agriculture and rural areas [2]. After China succeeded in lifting areas from poverty in 2020, it expanded its efforts by developing these areas further and by revitalizing rural areas [3]. These objectives mark new phase of the battle against poverty, entitled “agriculture, rural areas, and farmers” [4]. The county level of geography is a basic building block for human–land relations [5,6,7], and it is the basic unit of China’s national economy [8]. Because of this, China’s urban–rural development and rural revitalization use counties as the basic unit for development. However, as these efforts continue to advance, it has become clear that development has been unbalanced and insufficient in some counties, garnering interest from the government and academia. At present, China’s rural areas are shifting from the “blood-transfusion” stage of development, where material poverty was addressed, to the “blood-making” stage of development, where self-development is promoted [9,10]. This method will cultivate and grow the counties’ sustainable development capacity in multiple dimensions, including economic, social, environmental, and rural development [11,12,13], thereby narrowing the development gap between counties. As a result, China will be able to comprehensively promote rural revitalization and achieve common prosperity [14,15]. Therefore, to promote regional development and achieve rural revitalization goals, the government must first increase the counties’ sustainable development capacity.

County sustainable development capacity (CSDC) refers to the long-term, stable development of a county. Initially, this meant the pursuit of economic growth. However, CSDC has come to signify the coordinated and comprehensive development of a county’s economy, society, environment, and other dimensions [16,17]. Increasing sustainable development requires: (1) efficient and equitable human and financial investment, promoting intra- and inter-generational equity; (2) coordinating financial assistance with other supportive policies, and between groups receiving assistance and those who are not; (3) and modeling ways to build long-term, stable institutional mechanisms that promote revitalization, and then execute these plans to achieve the desired results [18,19].

Scholars have increasingly focused on endogenous regional sustainability, arguing that regional development is largely dependent on the resources and entrepreneurship that regions themselves possess [20,21,22,23,24,25]. The theoretical basis for this can be traced back to localization theory and the theory of regional self-reinvigoration. Maskell established the concept of localization theory in terms of institutions, resources, and skills, thus incorporating resource endowment and social innovation capacity into the study of regional development capacity [26]. Martin concentrated on endogenous growth theory and extended the study of endogenous revitalization capacity from the individual level to the household, firm, regional, and national levels, arguing that this self-revitalization capacity is related to the subject’s own ability to learn and innovate [27]. Finally, Sobczyk found that individuals are often able to create jobs and stimulate economic dynamism in the process of sustainable development, and that such sustainable development activities are especially beneficial in rural areas [28]. As a result, China’s CSDC utilizes theories of endogenous regional sustainability to achieve rural revitalization and increase county-level equity.

Researchers from China have established much of the foundational literature on methods to improve sustainable development capacity in less developed regions [29,30,31]. In a paper analyzing the economic development of ethnic minority regions, Xu made an early link between the economic development of poor regions and their sustainable development capacity [32]. Zheng et al., on the other hand, identified the problem of unbalanced county development, and believed that the economic development gap between east, central, and west China stems from gaps in county development [33]. Other scholars have attributed China’s inter-regional development imbalances to regional differences in sustainable development capacity, arguing that less developed regions must increase this capacity in order to achieve sustainable development [34,35,36]. Therefore, based on different perspectives, such as sustainability theory [37,38], self-development capacity theory [39,40], poverty theory [41,42], and human–land relationship theory [43,44], there have been numerous empirical studies on the sustainability of different research regions [45,46]. While these indicator systems represent varying theoretical foundations, they tend to focus on economic, environmental, and social facets of a region [47,48,49]. Despite this abundance of research, available studies have largely been conducted on a regional-scale, identifying macro-level trends as the impetus for lagging development [50]. As such, little is known about the county-scale, which could prove to be an important step in the path of rural revitalization.

China’s rural revitalization strategy focuses on counties as the primary unit in which to affect change for towns and villages, as well as their social and natural elements. Accordingly, improving the county’s sustainable development capacity is a basic requirement for the rural revitalization of the county. Central Document No. 1 of 2021 stated that “For counties that have escaped poverty, a five-year transition period will be established from the date of escaping poverty, so that they can be supported and on the right path”, specifically by “supporting localities to independently select some of the counties that have escaped poverty as key counties to receive assistance in pursuing rural revitalization”. In August of the same year, the National Administration for Rural Revitalization, in accordance with the decision of the “Opinions on Realizing the Effective Interface between Consolidating and Expanding the Results of Poverty Alleviation and Rural Revitalization”, identified 160 key counties to receive assistance in pursuing rural revitalization in China (hereafter referred to as “key counties to receive assistance”) in 10 western provinces (cities anddistricts). Additionally, each province selected key counties to receive assistance in pursuing rural revitalization according to its needs. The success and expansion of poverty eradication efforts and the promotion of rural revitalization on a county basis indicate that the theory has been well-integrated into policy. Despite this political and social progress, the available studies have largely focused on a single province or municipality directly under the central government, rather than examining the situation at the county-scale. As such, currently, there is no study that focuses on the sustainable development capacity of key counties to receive assistance in pursuing rural revitalization in China, nor explorations of decreasing the development gap between counties. What is the level of sustainable development capacity of the key counties to receive assistance, how to measure it, what are the specific deficiencies, and what are the underlying reasons for them? This is the core problem that this paper focuses on.

To fill this critical knowledge gap, this paper explores CSDC by constructing an evaluation system containing 29 indicators in three dimensions: economic aggregation capacity, social development capacity, and environmental carrying capacity. This evaluation system is then used explore the sustainable development capacity, development weaknesses, and the development gap between key counties to receive assistance. Finally, this paper expands the existing literature on regional sustainable development as follows: (1) This paper constructs a multi-dimensional and multi-level scientific CSDC score system and evaluates counties based on an accurate understanding of county sustainable development capacity and applying this system to a sample of 103 key counties to receive assistance in pursuing rural revitalization in China. (2) The level and spatial distribution of CSDC were visualized using ArcGIS 10.8, which clarifies the counties’ sustainable development capacity weaknesses and the development differences between regions. (3) This paper used SPSS cluster analysis to identify weaknesses restricting CSDC and classify the categories of counties to receive assistance, allowing critical counties to be targeted and nurtured. Ultimately, this paper can be used by policymakers to strengthen key counties to receive assistance by targeting resources to expand their CSDC, which in turn promotes sustainable development and rural revitalization. Further, linking poverty alleviation efforts with rural vitalization will create a solid foundation for poverty alleviation, making it more sustainable in the long run.

The rest of the study is structured as follows: Section 2 presents the data and methodology used in this paper; Section 3 examines the measurement results and spatial distribution of the sustainability scores and capacity scores for each dimension in the key counties to receive assistance and discusses the classification of restriction types; and Section 4 contains the conclusions and policy implications.

## 2. Materials and Methods

### 2.1. Construction of the Index System

Reference has been made to the method of constructing the indicator system of sustainable development capacity by scholars based on the theoretical connotation of county sustainable development capacity and in consideration of the availability of data [36,37,38,39]. The construction of the indicator system follows the principles of scientific, systematicity, scientificity, operability, comparability, and comprehensiveness. In this study, CSDC was deconstructed into three secondary subsystems: economic aggregation capacity, social development capacity, and environmental carrying capacities (EAC, SDC, and ECC, respectively). Additionally, indicators that are more representative of each dimension were selected to reflect the level of CSDC in this region (see Table 1).

EAC includes nine indicators. To be specific, “gross domestic product (GDP) per capita” and “economic density” reflect the whole benefit of county economic development; “the proportion of the tertiary industry” embodies the optimization of county industrial structure to some extent; “the per capita disposable income of urban residents” indicates the affluence level of the population; “the proportion of population with university education” shows educational attainment and potential for innovation; “road density” denotes the level of infrastructure; “labor density” represents the number of laborers per unit area and reflects the adequacy of labor supply; “the consumption ratio of rural and urban residents” refers to the ratio of per capita consumption expenditure between them, partly reflecting the economic gap and imbalance between rural and urban areas; and “the per capita agricultural, forestry, animal husbandry, and fishery production value in rural areas” measures the efficiency of agricultural production and reflects the modernization level of agriculture. 

SDC contains nine indicators, including urbanization and urban registered unemployment rates. Of these, “urbanization rate” reflects the urbanization level of the resident population; “urban registered unemployment rate” is an important indicator of the employment situation of the population; “the number of medical and health technicians per 1000 people” and “beds per 1000 people” measure the coverage level of local health care; “the degree of fiscal self-financing” gauges the relationship between local revenue and final local expenditure, reflecting the degree of fiscal self-reliance; “per capita fiscal expenditures on public services, social security, and employment as well as urban and rural communities” indicate the level of county social security; and “the number of rural people with minimum living security per 1000” shows the proportion of the low-income population.

ECC covers eight indicators. Of these, “the number of days with good air quality” and “annual precipitation” reflect the level of local climate and the livability level of the county; “land area per 10,000 inhabitants” describes ECC and development potential; “the proportion of grain-sown areas in the county” and “forest coverage rate” indicate the use and protection levels of land and forest resources; “per capita fiscal expenditure on energy conservation and environmental protection” measures the level of environmental governance; and “grain production potential”, i.e., grain yield, represents the use level of farmland resources and agricultural production.

### 2.2. Overview of the Study Area

**Figure 1:**
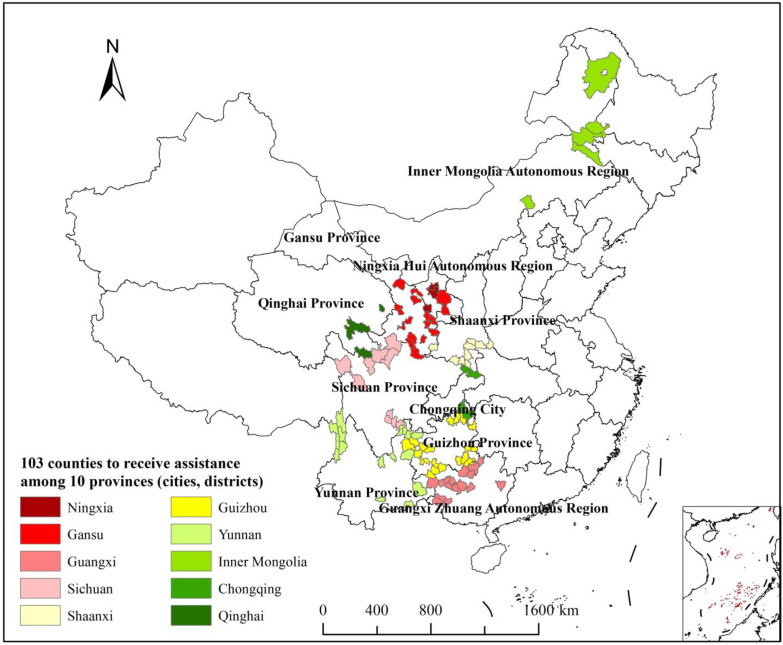
Study areas.

According to the list of key counties to receive assistance announced by the Chinese government in August 2021, the National Administration for Rural Revitalization expanded poverty eradication efforts focused on remote or high-altitude counties in the western region of China because the natural environment is relatively harsh and the economic base and social institutions are not well-developed. They entered the next phase of this effort by evaluating farming communities in the 10 western provinces (cities and districts) according to the follow indices: per capita GDP, per capita general public budget revenue, and per capita disposable income. Additionally, they integrated factors such as the timing of poverty alleviation and the risk of returning to poverty. This evaluation allowed 160 key counties to be identified as candidates to receive assistance in pursuing rural revitalization, which is distributed in Inner Mongolia Autonomous Region, Guangxi Zhuang Autonomous Region, Chongqing City, Sichuan Province, Guizhou Province, Yunnan Province, Shaanxi Province, Gansu Province, Qinghai Province, and Ningxia Hui Autonomous Region. A map of the spatial distribution of the 103 key counties is shown in Figure 1. See Appendix A for the specific list.

### 2.3. Data Sources

This paper considered the full coverage of sampling in each province (city and district), and statistics on a total of 27 indexes in three dimensions were obtained after screening for a total of 103 counties in the 10 western provinces (cities and districts) that are the focus of national rural revitalization support. The data mainly came from 2020 National Economic and Social Development Statistical Bulletin for each county, the 2021 China Rural Poverty Monitoring Report, the China County Statistical Yearbook, and the websites of the Data Centre for Resource and Environmental Sciences of the Chinese Academy of Sciences, the National Geomatics Center of China, and the statistical bureaus of each administrative unit. The breakdown of fiscal expenditure was taken from the public financial accounts of each county (district) people’s government; data on urban registered unemployment rate, forest coverage, annual precipitation, and number of days with good air quality were obtained from the Government Work Report. Data on the proportion of the population with university education, labor force density, and urbanization rate were obtained from the Seventh Population Census Bulletin. Data on grain sown area and grain production potential were obtained from the Wind database. Any missing data were filled in by the interpolation method [51,52,53].

### 2.4. Research Methods

The TOPSIS model based on AHP–entropy weights is, essentially, an improvement on the traditional TOPSIS evaluation method. The core idea of the TOPSIS method is to define the distance between the optimal and inferior solutions to a decision problem, and finally to calculate the relative paste progress of each solution to the ideal solution and to rank the solutions in order of merit [54,55,56].

Weight determination is a fundamental part of the empirical research in this paper. Previous similar evaluation studies have more often used principal component analysis, the AHP method, and entropy weighting method to determine the weights [57,58,59,60,61,62,63]. Analytic hierarchy process (AHP) refers to decomposing a complex multi-objective decision-making problem into multiple levels, and then conducting qualitative and quantitative analysis, which makes the evaluation results more systematic and hierarchical, but the influence of subjective factors is difficult to avoid [58]. The entropy method, as a type of objective assignment method, determines weights based on the degree of variation of each index. The greater the degree of variation in the entropy weight coefficient of the selected indicator, the higher the importance of the indicator [60]. It does not depend on the subjective attitude of the decision maker and the evaluation process is more reproducible. As a result, it is now widely used in the engineering, social, and economic fields, but it ignores the importance of the indices themselves, and sometimes the determined index weights are far from the expected results. Due to the one-sided nature of the single-power evaluation method and the above disadvantages, it tends to lead to some bias in the results of the weight calculation [61,62]. Therefore, the combination of the AHP and entropy power evaluation methods makes the obtained weighting coefficients more scientific and reasonable.

The main calculation steps of the AHP–Entropy TOPSIS method are as follows.

AHP is first used to determine the subjective weights.

(1) Construct the judgment matrix. According to method of two elements contrasting, indices at the same level are compared two by two and assigned a value based on the 1 to 9 comparison scale. *b_i_* and *b_j_* are used to represent evaluation indexes, *b_ij_* represents the importance of bi relative to *b_j_*, with *b_ji_* = 1/*b_ij_*, and the discriminant matrix *P* is obtained.

(2) Solve the weight and test consistency. 

After the discriminant matrix Ρ is obtained, ${Ρ\alpha }_{j}=\lambda_{max}\;\alpha_{j}$ is used to calculate $\alpha_{j}$ and $\lambda_{max}$, where $\alpha_{j}$ is the weight vector and $\lambda_{max}$ is the maximum eigenvalue. Then, accordingly:

(1) $$CI=\frac{\lambda_{max}-n}{n-1}$$

The random consistency index *RI* is used to calculate the consistency ratio *CR*. When *CR* < 0.1, the judgment matrix is considered to have passed the one-time test; otherwise, it should be modified again.

(2) $$CR=\frac{CI}{RI}$$

Secondly, the entropy weight method is used to determine the objective weight.

(3) The judgment matrix is constructed to normalize the original decision matrix. The indices for evaluating the CSDC are divided into positive and negative indices. The larger the value of the positive index, the higher the capacity level, and the smaller the value of the negative index, the higher the capacity level. For the comparability of the index data, dimensionless normalization is performed on it, and the processed index value is between [0, 1]. The positive index is calculated according to Formula (3) and the negative index is calculated according to Formula (4):

(3) $${x^{\prime }}_{ij}=\frac{x_{ij}-minx_{ij}}{maxx_{ij}-minx_{ij}}$$

(4) $${x^{\prime }}_{ij}=\frac{maxx_{ij}-x_{ij}}{maxx_{ij}-minx_{ij}}$$

where $x_{ij}$ represents the original value of the evaluation index and ${x^{\prime }}_{ij}$ is the standard value.

(4) Find the entropy information of the *j*th index.

(5) $$H_{j}=-\frac{1}{\ln m\sum_{i=1}^{m}p_{ij}\ln p_{ij}}(i=1,2\ldots ,m;j=1,2\ldots ,n)$$

where when *f_ij_* = 0, let *f_ij_*ln*f_ij_* = 0.

(5) Find the entropy weight of the *j*th index.

(6) $$W_{j}=\frac{1-H_{j}}{\sum_{j=1}^{n}\left(1-H_{j}\right)}$$

where $0\leq W_{j}\leq 1,\sum_{j=1}^{n}w_{j}=1$. The combined weights of the indices are then determined.

(6) In order to avoid the limitation of a single weighting, the assignments are combined by means of Formula (7), where $\alpha_{j}$ is the weight of the *j*th index obtained using AHP and *W_j_* is the weight of the *j*th index obtained using the entropy weighting method.

(7) $$\beta_{j}=\frac{w_{j}\times \alpha_{j}}{\sum_{j=1}^{n}\left(w_{j}\times \alpha_{j}\right)},j=1,2,3,...,n$$

Finally, a modified TOPSIS model is applied to calculate the capability score.

(7) Construct a weighted decision matrix *V*.

(8) $$V=\beta_{j}\times {x^{\prime }}_{ij}$$

(8) Determine the positive and negative ideal solutions for the indices. *V^+^* denotes the best of all solutions and is called the positive ideal solution; *V^−^* denotes the least ideal solution and is called the negative ideal solution.

(9) $$V^{+}=\left\{\max v_{ij}|i=1,2,\ldots ,m\right\}$$

(10) $$V^{-}=\left\{\max v_{ij}|i=1,2,\ldots ,m\right\}$$

(9) Calculate the Euclidean distance. The distances from each evaluation area vector to the positive and negative ideal solutions are set to *D^+^* and *D^−^*, respectively, as follows.

(11) $$D^{+}=\sqrt{\sum_{j=1}^{m}{\left(V_{ij}-V_{j}^{+}\right)}^{2}}(i=1,2,\ldots ,n)$$

(12) $$D^{-}=\sqrt{\sum_{j=1}^{m}{\left(V_{ij}-V_{j}^{-}\right)}^{2}}(i=1,2,\ldots ,n)$$

(10) Calculate the closeness *C_j_*.

(13) $$C_{j}=\frac{D^{-}}{D^{+}+D^{-}}$$

The closeness indicates how close the rating object is to the positive ideal solution, i.e., the optimal solution, which is denoted by *C_j_*. Clearly, the closer *C_j_* is to 1 when $C_{j}\subset (0,\;1)$, the closer the CSDC is to the optimal level and the higher the county sustainable development capacity; conversely, the closer *C_j_* is to 0, the further away CSDC is from the optimal level, i.e., the lower CSDC is, pending further improvement.

## 3. Results and Analysis

### 3.1. Calculating and Analyzing CSDC in Key Counties to Receive Assistance

The empirical analysis was performed via SPSS 26.0 to obtain the weights of each indicator (see Appendix B). The average score and ranking of CSDC in key counties to receive assistance in pursuing rural revitalization in each province (city and district) are shown in Table 2.

All 103 key counties to receive assistance in pursuing rural revitalization had an overall score of less than 0.5 in terms of sustainable development capacity, which differed significantly from the optimal solution. Three key counties to receive assistance in pursuing rural revitalization in Qinghai Province ranked in the upper-middle range overall, with an average score of 0.3873, ranking first among the ten provinces (cities, districts), which was followed by Chongqing City and Inner Mongolia Autonomous Region, where the average score for their CSDC in their areas was 0.3743 and 0.3694, respectively. Ningxia Hui Autonomous Region ranked at the bottom of the ten provinces (scities, districts), with the three key counties to receive assistance in pursuing rural revitalization in its territory scoring only 0.2723 for SDC. Additionally, the average score of CSDC in the key counties to receive assistance in pursuing rural revitalization in the three provincial areas of Inner Mongolia Autonomous Region, Yunnan Province, and Sichuan Province varied widely, showing a strong uneven and insufficient development.

Long et al. and Cheng et al. have established that rural revitalization is a development strategy to achieve economic, political, cultural, and ecological revitalization by reshaping the socio-economic form and spatial pattern of the territory [64,65]. This study found scores and spatial patterns of key counties to receive assistance that are similar to those of Dong et al., who found a strong spatial relationships whereby multidimensional poverty increased from the eastern to western regions [41]. Consistent with Qi et al., the visualization results demonstrate that the northwestern region generally had a low score in sustainability, indicated by the large differences between provinces [66]. Qinghai Province was an exception to this trend, and it scored high in sustainability due to specific geographic factors.

**Figure 2:**
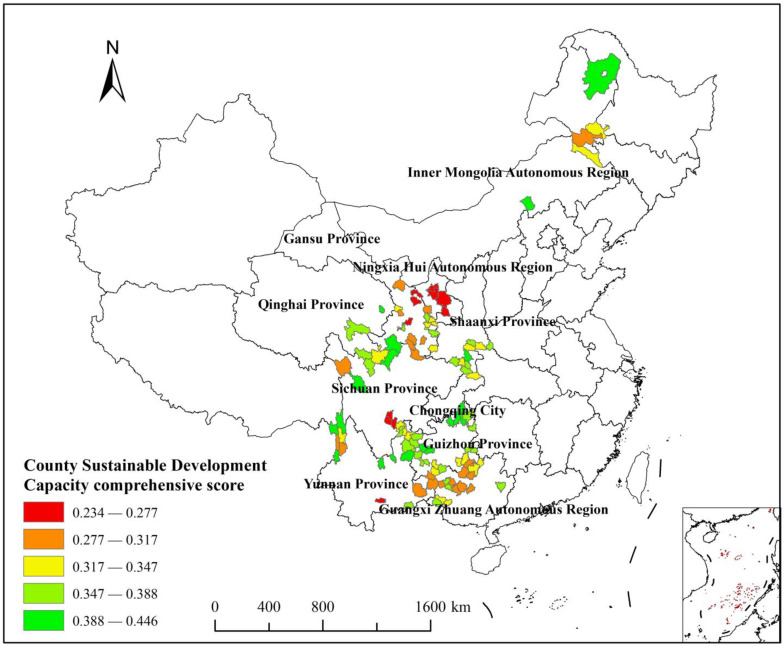
Classification result of CSDC comprehensive score of key counties to receive assistance.

The CSDC scores of the key counties to receive assistance were portrayed (see Figure 2) using ArcGIS 10.8. At the same time, the county sustainability score was classified into five levels: low, medium-low, medium, medium-high, and high, with reference to the Natural Breaks Classification method [67] (see Table 3).

This study found that most of the 103 key counties to receive assistance in pursuing rural revitalization are within in the borders of the 10 western provinces (scities, districts). These counties had lower scores in economic aggregation, social development, and environmental carrying capacity than other regions. Moreover, more than half of the CSDC scores were in the low to medium tier type, which is well below the optimum.

Of the 103 key counties to receive assistance, the results of the score classification, based on their sustainable development capacity, are as follows: ten counties with low values in sustainable development capacity (0.23400 ≤ CSDC < 0.27700), most of which have major concentrations of ethnic minorities in China. The key counties to receive assistance, occupying the largest number of them, were located in Gansu Province in the northwest, followed by Sichuan Province and Ningxia Hui Autonomous Region with three and two, respectively, while Honghe County in southern Yunnan Province, with a score of only 0.27, was also ranked among them. Twenty-two counties with low to medium values in SDC (0.27701 ≤ CSDC < 0.34700) were mainly distributed in Guangxi Zhuang Autonomous Region, Gansu Province, and Yunnan Province. Twenty-four counties had a median SDC (0.34701 ≤ CSDC < 0.38800), of which Gansu Province, Guangxi Zhuang Autonomous Region, Guizhou Province, and Shaanxi Province all hold for four. There were twenty-eight counties with scores in the medium-high range for SDC (0.34701 ≤ CSDC < 0.38800), mainly concentrated in nine counties in Guizhou Province, with four counties in Guangxi Zhuang Autonomous Region, Shaanxi Province, and Yunnan Province. In addition, there were nineteen counties with a high score for SDC (0.38801 ≤ CSDC < 0.44600), mostly located in the multi-provincial convergence zone with more opportunities for exchange and input of resources with the outside world, mainly concentrated in the counties of the Yunnan, Guizhou, and Sichuan regions in southwestern China. The two counties with the greatest CSDC scores had stronger comprehensive endogenous development dynamics than the other counties. The Zhengxiangbai Banner in the Inner Mongolia Autonomous Region was ranked the most highly. This area is next to the Great Grassland and connects the eastern Inner Mongolia and western Inner Mongolia economic zones from east to west. Additionally, it is one of the closest areas in the Inner Mongolia Autonomous Region to the Bohai Economic Circle, which is an important ecological barrier in Beijing and Tianjin, an important new green industrial base in the south of Ximeng, and an important node and distribution center for trade and logistics. The second highest ranking county is Gongshan, Nujiang, which has an alpine valley terrain that includes Deep in The Clouds Mountain, Gaoligong Mountain, Dandanglika Mountain, Salween River, and Dulong River running through the county. It has a temperate climate; is rich in mineral, biological, and soil resources; and is home to 20 ethnic minorities, including the Lisu, Nu, Bai, and Naxi, creating a diverse culture. Key counties to receive assistance in pursuing rural revitalization have advantages in education, media, social welfare, and health care.

### 3.2. Calculation and Analysis of the Secondary Indices of CSDC

**Figure 3:**
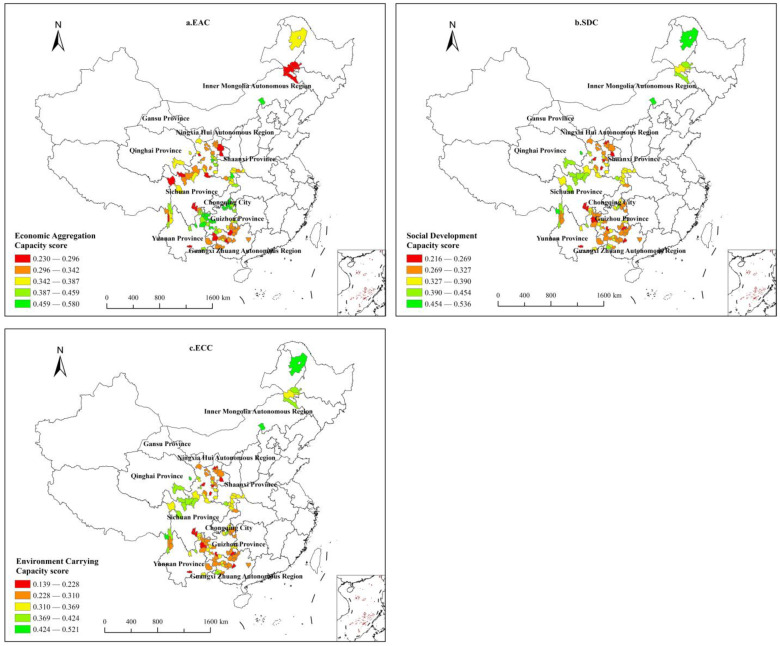
CSDC score of key counties to receive assistance according to the secondary indices.

This study measured the following as secondary indices of CSDC: economic aggregation capacity (EAC), social development capacity (SDC), and environmental carrying capacity (ECC). Each of these dimensions had their calculated scores split into five levels: low, medium-low, medium, medium-high, and high, with reference to the Natural Breaks Classification method. Additionally, visualization was carried out via ArcGIS 10.8 (Figure 3).

#### 3.2.1. Economic Aggregation Capacity (EAC) Score

Eighteen counties were identified as having a low EAC score (0.23000 ≤ EAC < 0.29600) and were primarily found in Gansu Province, Sichuan Province, Guangxi Zhuang Autonomous Region, the Inner Mongolia Autonomous Region, and other provinces or regions with a high concentration of minority communities. Twenty-nine counties had low-medium scores (0.29601 ≤ EAC < 0.34200), which was the greatest number of counties in a category. These counties were concentrated in Guangxi Zhuang Autonomous Region, Gansu Province, and Sichuan Province. Twenty-two counties had EAC scores in the medium range (0.34201 ≤ EAC < 0.38700), and were concentrated in Gansu, Shaanxi, and Guizhou Provinces. Eighteen counties had EAC scores in the medium-high range (0.38701 ≤ EAC < 0.45900); six counties were in Guizhou, four were in Yunnan Province, and four were in Guizhou Province. Sixteen counties had EAC scores in the high range (0.45901 ≤ EAC < 0.58000); eight counties were in Guizhou Province and four counties were in Yunnan Province. The top-ranked city, Xuanwei, had a score of 0.58. It is in the northeastern part of the Yunnan Plateau, which slopes and transitions to Guizhou Plateau. This location has convenient transportation, superior resources, and a leisure resort, all of which attract tourists. These qualities afford this county a stable economic foundation on which to promote county sustainable development capacity.

#### 3.2.2. Social Development Competency (SDC) Score

Thirteen counties had a low value for SDC (0.21600 ≤ SDC < 0.26900), mainly located in Gansu, Guizhou, and Sichuan provinces; thirty-eight counties had a low value for SDC (0.26901 ≤ SDC < 0.32700), which was the type with the largest number of counties, concentrated in the northern area of Guangxi Zhuang Autonomous County, followed by Guizhou Province, including nine counties; thirty-two counties (0.32701 ≤ SDC < 0.39000) had a median value for SDC, mainly in the southern Shaanxi Province, southwestern Guizhou Province, and southern Gansu Province; sixteen counties had a medium-high value for SDC (0.39001 ≤ SDC < 0.45400), which were mainly distributed in Sichuan Province and Yunnan Province. Only four counties had a high value for SDC (0.45401 ≤ SDC < 0.53600), namely Zhengxiangbai Banner and Oroqen Autonomous Banner in Inner Mongolia Autonomous Region, Jianzha County in Qinghai Province, and Gongshan Dulong Nu Autonomous County in Yunnan Province. Among them, Oroqen Autonomous Banner is located in the northeast of Hulunbuir City, which is in the southern piedmont region of Daxing’ anling Mountain and on the west bank of the Nenjiang River. This county experiences significant seasonal changes, has access to abundant natural resources, and has cultivated well-developed social welfare programs. Jianzha County was ranked first in its SDS score out of 103 counties because its medical care, education, culture, social security, infrastructure, and other social undertakings have developed prominently. This county is located in the southeastern part of Qinghai Province, a vast and sparsely populated area. The region has a strong religious and cultural heritage because it is the birthplace to the second movement of Tibetan Buddhism.

#### 3.2.3. Environmental Carrying Capacity (ECC) Score

Ten counties had a low value for ECC (0.13900 ≤ ECC < 0.22800), namely seven counties in Gansu Province and two counties in Ningxia Hui Autonomous Region and Yuexi County in Sichuan Province, which are mostly located in remote or high-altitude areas with relatively harsh natural environment in the western region of China. Eighteen counties had a low-medium value for ECC (0.22801 ≤ ECC < 0.31000), with Gansu Province still having the highest number at eight, Inner Mongolia Autonomous Region, Sichuan Province and Yunnan Province each having three, and Xiji County in Ningxia Hui Autonomous Region, which was in the heart of the Loess Plateau, also being listed. Twenty-six counties had a median value for ECC (0.31001 ≤ ECC < 0.36900), with the highest number in Yunnan, Guizhou, and Shaanxi Provinces. Thirty-six counties had medium-high values for ECC (0.36901 ≤ ECC < 0.42400), which was the most concentrated fractional segment, with fourteen counties in Guizhou Province ranking among them, followed by nine counties in Guangxi Zhuang Autonomous Region and four counties in Shaanxi Province and Yunnan Province. Thirteen counties had high values (0.42401 ≤ ECC < 0.52100), including four provinces in Guangxi Zhuang Autonomous Region, three counties in Yunnan Province, and two counties in Sichuan Province. In addition, the Oroqen Autonomous Banner of the Inner Mongolia Autonomous Region, Langao County of Shaanxi Province, Zhaoping County of Guangxi Zhuang Autonomous Region, Pengshui County of Chongqing City, and Nayong County of Guizhou Province had the highest ECC scores out of the 103 key counties to receive assistance because they have abundant resources.

#### 3.2.4. Comparison to Previous Studies

The results reveal that, consistent with previous findings [36,44], economic indicators are an important component of the economic–social–environmental evaluation system; however, our results differ from other studies because the CSDC evaluation system this study constructed afforded a weight of 31.82% to EAC, 33.80% to SDC, and 34.37% to ECC (see Appendix B). That is because ECC, in particular, is an important component in evaluating sustainable development capacity, which is consistent with previous findings [46,48,50].

The findings by Fazlagić and Skikiewicz [36] and Fei et al. [44] suggest that, when evaluating the EAC dimension, consumption and income per capita hold a greater weight. Conversely, our study found that economic density, road density, and labor density held a greater weight. Within the SDC dimension, the degree of self-financing held the greatest weight, followed by fiscal expenditure on public services and urban and rural communities, and, in line with the findings of many studies, health indicators [68]. For the ECC dimension, forest coverage rate, air quality, and tourism development were all important, which is consistent with previous studies [69,70,71].

The spatial patterns of the overall CSDC scores and the scores of its secondary indicators demonstrate that the sustainable development capacity of each region is the result of a multidimensional synergy; differences in the economic base, community governance, and environmental conditions of each region led to major differences in the spatial distribution of the secondary indicators. These results illustrate why it is particularly important to identify the weaknesses of counties with lower overall CSDC scores and secondary indicator scores so that efforts can be focused on targeting gaps in sustainable development and formulating rural revitalization policies according to local conditions.

### 3.3. Identification and Classification Evaluation of the Weakness of CSDC

**Figure 4:**
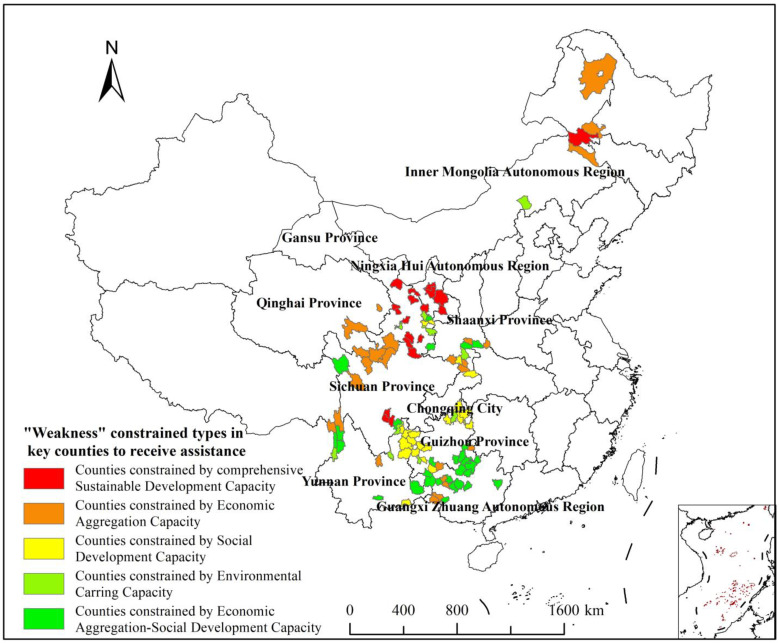
Distribution of the “weakness”-constrained types in 103 key counties to receive assistance.

This study identified the development weakness of the 103 key counties to receive assistance by using a K-means cluster analysis for their EAC, SDC, and ECC scores, and utilized the Euclidean distance as the similarity index to minimize the sum of squares of the clusters [72]. There was no clear indicator of the similarity among the data, so the number of clusters was not clearly specified in the cluster analysis. Therefore, this study followed the principle of consistency of dominant factors [48], and chose five “weakness”-constrained types according to the cluster analysis hierarchical clustering spectrum diagram. There were: types constrained by comprehensive CSDC (counties in category I), types constrained by EAC (counties in category II), types constrained by SDC (counties in category III), types constrained by ECC (counties in category IV), and types constrained by Economic Aggregation–Social Development Capacity (counties in category V). These types, respectively, accounted for 17.48%, 24.27%, 20.39%, 9.71%, and 28.16% of the key counties to receive assistance. The basic characteristics statistics are shown in Table 4 and their spatial distribution is shown in Figure 4. See Appendix C for the list of specific classifications.

This paper determined that EAC was an important index for measuring CSDC because a total of 72 (69.9%) key counties to receive assistance in categories I, II, and V had EAC scores that restrict the development of the county, which in turn limits improvement in other dimensions of development capacity. This bottleneck could result in a multi-factor “weakness” that constrains their overall CSDC. In this way, our model predicted the reality of these counties: counties in categories II, III, and IV have single-factor constraints or an obvious “weakness” that constrains their ability to improve their overall sustainable development, despite having a good foundation for development in other dimensions.

Key counties to receive assistance in category I were constrained by their comprehensive CSDC. The lowest-scoring counties in this category were mostly in Gansu Province (eleven counties), followed by Ningxia Hui Autonomous Region (three counties) and Sichuan Province (three counties). The average score of their CSDC was 0.2782, which is far lower than any of the other counties. Furthermore, the scores of their secondary indices (EAC, SDC, and ECC) were also the lowest. Many of these counties are home to ethnic minorities and are located in the fringe areas of larger provinces (cities, districts). In general, the foundation of their economic development is weak, the development of social services is relatively lagging, the environmental conditions are poor, and the rural area development is delayed. Moreover, many of these counties are located in desert areas and plateau alpine regions, which have a fragile natural ecology and lack adequate transportation and other infrastructure. Thus, their low CSDC is constrained by various factors. Although these counties have been lifted out of poverty, they need the most attention and multi-dimensional support because their endogenous development is lacking.

Key counties to receive assistance in category II were constrained by EAC, and were distributed across all provinces (cities, districts), except Ningxia Hui Autonomous Region. They were mainly distributed in Sichuan Province (five counties), Guangxi Zhuang Autonomous Region (four counties), and Shaanxi Province. (four counties), a total of twenty-five counties. The average score of the CSDC in this type of counties was 0.3804, higher than the average of the entire sample. With the exception of the average score of EAC being lower than the overall average level, the other two-dimensional secondary indices, SDC and ECC, were higher than the overall average level. These counties have relatively complete medical facilities and personnel; attach great importance to social services, such as culture, media, and tourism; and have a sustainable ecological environment, good agricultural production conditions, and a minimal amount of rural residents. However, the lack of market vitality, inconvenient transportation, weak educational infrastructure, and a net outflow of the labor force hinder the ability of these key counties to receive assistance to coordinate economic development and agglomeration. In turn, this constrains the development of other fields, holding back their comprehensive capabilities. Therefore, it is necessary to focus on increasing resource input and support for the economies of counties in category II.

Key counties to receive assistance in category III were types constrained by SDC, and were mainly concentrated in Guizhou Province (ten counties), Yunnan Province (five counties), and Chongqing City (three counties), with a total of twenty-one counties. The average score of CSDC in this type of counties was 0.3703, which was higher than the average of the entire sample. With the exception of the average score of SDC, which was lower than the overall average level, the average scores of other two-dimensional secondary indexes, namely EAC and ECC, were at a relatively high level. SDC was a significant “weakness” that constrained the CSDC. Their financial self-sufficiency is poor, medical resources are scarce, and various financial expenditures—especially in the culture, sports, tourism, and media—are lacking. Counties in this category require the government to pay attention to social and livelihood conditions, such as employment security, public services, health care, and cultural tourism, and increase financial investment in public goods and services.

Key counties to receive assistance in category IV were types constrained by ECC, including three counties in Gansu Province, three counties in Yunnan Province, two counties in Guizhou Province, and Zhengxiangbai Banner in Inner Mongolia Autonomous Region and Hanbin District in Shaanxi Province, a total of ten counties. The CSDC score in this type was 0.3953, which was higher than the average of the entire sample. Their EAC and SDC had high scores, but their ECC score was lower than the overall average, which was a significant “weakness” that constrained CSDC. Counties in this type have harsh environmental conditions, are in remote areas, have a small area per capita, and have low forest coverage and rainfall. Some of these counties even have year-round droughts. These counties experience severe resource restrictions, so their tourism revenue and investment in energy conservation and environmental protection are relatively low. Therefore, the government should focus on addressing environmental issues, vigorously promoting ecological development, increasing residents’ energy conservation awareness and environmental protection, developing environment-friendly industries, and gradually expanding the supply capacity and environmental carrying capacity of ecological products and services in these counties.

Key counties to receive assistance in category V were constrained by Economic Aggregation–Social Development Capacity, which had the greatest number of counties and are concentrated in southwestern China. The province with the largest number was Guangxi Zhuang Autonomous Region (twelve counties), followed by Guizhou Province (six counties) and Yunnan Province (five counties), with a total of twenty-nine counties. The average score of CSDC of this type was 0.3177, which was lower than the average score of the entire sample. This type has a good environmental resource base, vast forest coverage, sufficient precipitation, and great potential for developing tourism industries. However, in these counties, there is a large gap between the income and consumption of urban and rural areas; the output of agriculture, forestry, animal husbandry, and fishery industries is relatively low; the proportion of people living in rural areas who have low incomes is large; the supply of public goods and services is insufficient; and a vast swath of the region is still relatively impoverished. As a result, this type of county has been the top priority for the expansion of poverty alleviation in recent years, and has required continuous promotion of rural revitalization. In these areas, therefore, the government should place more emphasis on developing medical institutions, investing in educational resources, and promoting the high-quality development of county agriculture, all of which will improve the well-being of the local populations and the long-term sustainable development of their economies.

## 4. Conclusions and Recommendations

### 4.1. Research Conclusions

This paper used the TOPSIS method based on AHP–entropy weight to calculate the CSDC of 103 key counties to receive assistance in pursuing rural revitalization from three dimensions in the year 2020, namely economy, society, and environment, and conducted a spatial analysis via ArcGIS 10.8. The following conclusions were drawn.

(1) The sustainable development capability of a region is the product of the synergistic interaction of economic, social, and environmental multidimensional factors. In addition to the economic factors that are traditionally perceived as dominant factors, the environment also plays a considerable role. The overall CSDC score in key counties to receive assistance in pursuing rural revitalization in China was low, which is reflected by the deficiency of secondary indicator capacity, the incongruity of EAC, SDC, and ECC, or the existence of “weaknesses”.
(2) According to CSDC score, 103 counties could be divided into five categories, including nineteen counties with a high value, twenty-eight counties with a medium-high value, twenty-four counties with a medium value, twenty-two counties with a medium-low value, and ten counties with a low value. In contrast, Ningxia Hui Autonomous County and Gansu Province, which are in the northwest region, had a low CSDC score. Their development has been constrained by historical, regional, economic, cultural, and religious factors. Additionally, their local governments are inexperienced and incapable of managing economic and social development. Coupled with the fragile ecology and inaccessibility, counties in these regions do not have a strong potential for sustainable development.
(3) Cluster analysis by EAC, SDC, and ECC identified the 103 key counties to receive assistance in pursuing rural revitalization into five constrained types, including eighteen counties constrained by comprehensive CSDC (counties in category I), twenty-five counties constrained by EAC (counties in category II), twenty-one counties constrained by SDC (counties in category III), ten counties constrained by ECC (counties in category IV), and twenty-nine counties constrained by Economic Aggregation–Social Development Capacity (counties in category V), accounting for 17.48%, 24.27%, 20.39%, 9.71%, and 28.16%, respectively. The five different types of key counties to receive assistance that this study established represent five weaknesses in development capacity. Therefore, it is imperative to formulate support policies that correspond to the specific weaknesses in each category in order to promote the further development of these counties.

### 4.2. Policy Suggestions

The following policy recommendations are made in response to the above findings to implement targeted measures for different “weakness”-constrained types in key counties.

(1) Key counties to receive assistance in category I have shortcomings in several dimensions, such as EAC, SDC, and ECC, with insufficient endogenous development momentum and a low comprehensive development capacity. This category of counties should, first of all, further improve their social security system to prevent poverty from returning. In the meantime, policy and investment support for this category of counties should be strengthened concerning the development of industries enriching the people, the supply of public goods and services, and the improvement of the ecological environment. In addition, an innovative one-to-one support mechanism of “leading the weak by the strong” is required to form a multi- and all-around support and collaboration system.
(2) Key counties to receive assistance in category II should lay emphasis on optimizing their business environment and stimulating their economic development vitality because of a weaker EAC. Therefore, special industries should be developed based on county endowments and advantages. The purpose is to accelerate the transformation and upgrading of conventional industries, encourage the development of new industries and business models in rural areas, and build a system for the integrated development of primary, secondary, and tertiary industries. Additionally, it is important to adhere to the principle of intensification and market orientation, integrate all the existing types of parks in counties and create a county economic aggregation carrier. Meanwhile, the business environment in counties should be optimized to boost confidence in social capital investment and enhance the activity of social capital.
(3) Key counties to receive assistance in category III are lacking in sufficient SDC, whose priority is to upgrade the level of infrastructure and public service provision in counties. Continuously increasing investment in infrastructure and public services in this category of counties is necessary, especially in key areas such as transportation, water conservancy, electricity and education, healthcare, and elderly care. Additionally, ongoing efforts need to be made on the precision and inclusiveness of infrastructure and public service provision. Moreover, it is necessary to promote innovative pathways and strengthen technological empowerment. Furthermore, the initiative should be taken to connect with high-quality public service resources in cities through project cooperation and innovative forms such as “medical and educational communities”. By this means, the gap between rural and urban public services can be narrowed.
(4) Key counties to receive assistance in category IV have relatively low resources and ECC, and their focus should thus be on the simultaneous implementation of multiple measures to improve the rigid constraints of resources and the environment. Counties in this category should coordinate economic development with resource and environmental protection and develop strict resource protection and ecological compensation mechanisms with a view to focusing on the development of environmentally friendly industries. In parallel, the state should strengthen its “spatial support” for these counties and propel the interchange of resources and complementary advantages between them and regions with better resources and environmental conditions.
(5) Key counties to receive assistance in category V are weak in both EAC and SDC. The focus should be placed on the development of advantageous special industries on the basis of optimizing the supply of infrastructure and public services. In addition, infrastructure and public services should be continuously upgraded to strengthen the basic support for the industrial development of counties. Moreover, they should develop cultural tourism and advantageous special industries by focusing on their good resources and environment, and push forward the high-quality development of green agriculture with the aim of transforming ecological resources into ecological assets.

Based on the results of the above study, it is meaningful and remarkable to strengthen multidimensional support for key counties to receive assistance in pursuing rural revitalization. Firstly, it is beneficial to strengthening and extending the victorious achievements of China’s poverty eradication efforts. Secondly, it could make a significant contribution to continuing to promote the rural revitalization strategy, promoting common prosperity among farmers and preventing large-scale poverty return.

There are some shortcomings to this research. Firstly, due to the availability of data, only 103 counties out of 160 key counties to receive assistance were selected as the sample for this study. Therefore, this study was unable to address the entirety of the nation’s development. Secondly, 2020 was a crucial year for China to move from its efforts in poverty eradication to rural revitalization. This paper only collected, processed, and analyzed statistical data from the selected study counties for the year 2020, which may affect the accuracy of the findings because the data were limited. Thirdly, this paper was unable to capture all of the factors affecting CSDC, so the indicators selected in this paper may have imperfections, which in turn affects the reliability of the results. In future studies, we hope to continue to deepen and improve the research in this filed with richer theoretical knowledge and more comprehensive data.

## Figures and Tables

**Table 1:** Index system of the county sustainable development capacity.

Primary Index	Secondary Index	Tertiary Index (Units)	Index Calculation	Index Direction
County Sustainable Development Capacity (CSDC)	Economic Aggregation Capacity (EAC)	GDP per capita (CNY)	County GDP/permanent resident population	positive
		Economic density (CNY100 million/km^2^)	County GDP/county area	positive
		Proportion of tertiary industry	Proportion of tertiary industry	positive
		per capita disposable income of urban residents (CNY)	per capita disposal income	positive
		Proportion of population with university education	Population with university education/permanent resident population	positive
		Road density (km/100 km^2^)	Total mileage of county roads/county area	positive
		Labor density (person/km^2^)	Number of county labor force/county area	positive
		Consumption ratio of urban and rural residents	Per capita consumption expenditure of urban residents/per capita consumption expenditure of rural residents	negative
		Per capita agricultural, forestry, animal husbandry, and fishery production value in rural areas (CNY)	County agriculture, forestry, husbandry, and fishery gross output value/population of permanent rural residents	positive
	Social Development Capacity (SDC)	Urbanization rate	Urban permanent population/permanent resident population	positive
		Urban registered unemployment rate	Urban registered unemployment rate	negative
		Number of medical and health technicians per thousand people	Number of medical and health technicians in the county/permanent resident population × 1000	positive
		Beds per thousand people	County bed number/permanent resident population × 1000	positive
		Degree of fiscal self-financing	Local general public budget revenue/local general public budget expenditure	positive
		Per capita fiscal expenditure on public service (CNY)	Government expenditure on public service/permanent resident population	positive
		Per capita fiscal expenditure on social security and employment (CNY)	Government expenditure on social security and employment/permanent resident population	positive
		Per capita financial expenditure on urban and rural communities (CNY)	Government expenditure on urban and rural communities/permanent resident population	positive
		Number of rural people with minimum living security per thousand	Number of rural people with minimum living security/permanent resident population × 1000	negative
	Environmental Carrying Capacity (ECC)	Number of days with good air quality	Air quality excellent rate × 365	positive
		Land area per 10,000 people (km^2^)	County area/permanent resident population*10,000	positive
		Proportion of grain-sown areas in the county	County grain-planted areas/county area	positive
		Forest coverage rate	Forest coverage rate	positive
		Annual precipitation (mm)	Annual precipitation	positive
		Per capita tourism income (CNY)	County tourism income/permanent resident population	positive
		Per capita fiscal expenditure on energy conservation and environmental protection (CNY)	Government expenditure on energy conservation and environmental protection/permanent resident population	positive
		Grain production potential (t/HA)	Total county grain output /county grain-planted areas	positive

Note: Data for the county resident population, urban resident population, and rural resident population were all derived from data from the 7th census in 2020.

**Table 2:** Average sustainable development capacity score of key counties to receive assistance in each province (city and district).

Province (City, District)	Number of Key Counties	Average Score of CSDC of Key Counties	Ranking
Qinghai Province	3	0.3873	1
Chongqing City	4	0.3743	2
Inner Mongolia Autonomous Region	5	0.3694	3
Yunnan Province	16	0.3665	4
Guizhou Province	20	0.3635	5
Shaanxi Province	9	0.3566	6
Sichuan Province	10	0.3321	7
Guangxi Zhuang Autonomous Region	16	0.3246	8
Gansu Province	17	0.3105	9
Ningxia Hui Autonomous Region	3	0.2723	10

**Table 3:** Classification result of the sustainable development capacity comprehensive score of key counties to receive assistance.

Grade Category	Score Ranges	Quantity (pcs)	Distribution Area
low value	0.23400–0.27700	10	Gansu Province (4), Sichuan Province (3), Ningxia Hui Autonomous Region (2), Honghe County, Yunnan Province
medium-low value	0.27701–0.31700	22	Guangxi Province (8), Gansu Province (6), Yunnan Province (3)
medium value	0.31701–0.34700	24	Gansu Province (4), Guangxi Province (4), Guizhou Province (4), Shaanxi Province (4)
medium-high value	0.34701–0.38800	28	Guizhou Province (9), Guangxi Province (4), Shaanxi Province (4), Yunnan Province (4)
high value	0.38801–0.44600	19	Yunnan Province (6), Guizhou Province (5), Sichuan Province (3), Inner Mongolia Autonomous Region (2)

**Table 4:** The classification of “weakness” restriction types and the average value for each ability of key counties to receive assistance.

Types of “Weakness” in Key Counties to Receive Assistance	Quantity (pcs)	Average Score of CSDC	Average Score of EAC	Average Score of SDC	Average Score of ECC
Counties in category I	18	0.2782	0.3019	0.2908	0.2276
Counties in category II	25	0.3804	0.3440	0.4133	0.4075
Counties in category III	21	0.3703	0.4685	0.3161	0.3632
Counties in category IV	10	0.3953	0.4658	0.408	0.3304
Counties in category V	29	0.3177	0.3281	0.3021	0.3802
Five types of key counties to receive assistance	103	0.3443	0.3694	0.3403	0.3519

## Data Availability

Data are available from the corresponding author upon demand.

## Appendix A. List of 103 Counties with Key Rural Revitalization Assistance in China

**Province**	**Qty**	**List of Key Counties to Receive Assistance in Pursuing Rural Revitalization**
Inner Mongolia	5	Oroqen Autonomous Banner, Horqin Right Front Banner, Horqin Right Middle Banner, Zhalaite Banner, Zhengxiang White Banner
Guangxi	16	Rongshui Miao Autonomous County, Sanjiang Dong Autonomous County, Debao County, Lingyun County, Tianlin County, Jingxi City, Zhaoping County, Fengshan County, Donglan County, Luocheng Mulam Autonomous County, Huanjiang Maonan Autonomous County, Bama Yao Autonomous County, Du’an Yao Autonomous County, Dahua Yao Autonomous County, Xincheng County, Tianxian County, etc.
Chongqing	4	Chengkou County, Wuxi County, Youyang Tujia and Miao Autonomous County, Pengshui Miao and Tujia Autonomous County
Sichuan	10	Rangtang County, Aba County, Ruoergai County, Hongyuan County, Xinlong County, Dege County, Yuexi County, Ganluo County, Meigu County, Leibo County
Guizhou	20	Shuicheng District, Zheng’an County, Wuchuan Gelao and Miao Autonomous County, Guanling Buyi and Miao Autonomous County, Ziyun Miao and Buyi Autonomous County, Zhijin County, Nayong County, Weining Yi, Hui and Miao Autonomous County, Hezhang County, Yanhe Tujia Autonomous County, Songtao Miao Autonomous County, Qinglong County, Wangmo County, Ceheng County, Jinping County, Jianhe County, Rongjiang County, Congjiang County, Luodian County, Sandu Shui Autonomous County
Yunnan	16	Dongchuan District, Xuanwei City, Daguan County, Yongshan County, Zhenxiong County, Yiliang County, Ninglang Yi Autonomous County, Lancang Lahu Autonomous County, Wuding County, Honghe County, Maguan County, Guangnan County, Lushui City, Fugong County, Gongshan Dulong Nu Autonomous County, Lanping Bai and Pumi Autonomous County, Deqin County, Weixi Lisu Autonomous County
Shaanxi	9	Lueyang County, Zhenba County, Hanbin District, Ziyang County, Langao County, Shangnan County, Shanyang County, Zhen’an County, Zhashui County
Gansu	17	Jingyuan County, Maiji District, Qin’an County, Zhangjiachuan Hui Autonomous County, Gulang County, Zhuanglang County, Jingning County, Huan County, Zhenyuan County, Weiyuan County, Wen County, Dangchang County, Xihe County, Li County, Yongjing County, Dongxiang Autonomous County, Lintan County, Zhouqu County
Qinghai	3	Jianzha County, Maqin County, Banma County
Ningxia	3	Hongsipu District, Tongxin County, Xiji County

## Appendix B. AHP–Entropy Weight Method Used to Calculate the Index Weight

**Primary** **Index**	**Secondary** **Index**	**Combined Weight**	**Tertiary Index**	**Combined Weight**
County Sustainable Development Capacity (CSDC)	Economic Aggregation Capacity (EAC)	31.82%	GDP per capita (CNY)	3.08%
			Economic density (CNY 100 million/km^2^)	4.63%
			Proportion of tertiary industry	2.66%
			Per capita disposable income of urban residents (CNY)	2.69%
			Proportion of population with university education	3.59%
			Road density (km/100 km^2^)	5.71%
			Labor density (person/km^2^)	4.81%
			Consumption ratio of urban and rural residents	1.89%
			Per capita agricultural, forestry, animal husbandry, and fishery production value in rural areas (CNY/person)	2.76%
	Social Development Capacity (SDC)	33.80%	Urbanization rate	2.83%
			Urban registered unemployment rate	1.88%
			Number of medical and health technicians per thousand people	3.48%
			Beds per thousand people	2.48%
			Degree of fiscal self-financing	4.89%
			Per capita fiscal expenditure on public service (CNY)	4.16%
			Per capita fiscal expenditure on social security and employment (CNY)	5.06%
			Per capita financial expenditure on urban and rural communities (CNY)	3.52%
			Number of rural people with minimum living security per thousand	4.17%
			Degree of fiscal self-financing	1.33%
	Environmental Carrying Capacity (ECC)	34.37%	Number of days with good air quality	2.54%
			Land area per 10,000 people (km^2^)	5.25%
			Proportion of grain-sown areas in the county	4.64%
			Forest coverage rate	6.00%
			Annual precipitation (mm)	3.09%
			Per capita tourism income (yuan)	6.01%
			Per capita fiscal expenditure on energy conservation and environmental protection (CNY)	4.81%
			Grain production potential (t/HA)	2.03%

## Appendix C. Specific List of Five “Weakness”-Constrained Types

**Types of “Weakness” in Key Counties to Receive Assistance**	**Province**	**Prefecture Level City**	**County**
Counties constrained by integrated sustainable development capacity (counties in category I)	Inner Mongolia	Hinggan League	Horqin Right Front Banner
	Sichuan	Liangshan Prefecture	Yuexi County
			Ganluo County
			Meigu County
	Gansu	Baiyin City	Jingyuan County
		Wuwei City	Gulang County
		Qingyang City	Huanxian County
			Zhenyuan County
		Dingxi City	Weiyuan County
		Longnan City	Wenxian County
			Dangchang County
			Xihe County
		Linxia Hui Autonomous Prefecture	Yongjing County
			Dongxiang Autonomous County
		Gannan Prefecture	Zhouqu County
	Ningxia	Wuzhong City	Hongsipao District
			Tongxin County
		Guyuan City	Xiji County
Counties constrained by economic agglomeration capacity (counties in category II)	Inner Mongolia	Hulun Buir	Oroqin Autonomous Banner
		Hinggan League	Horqin Right Middle Banner
			Jalaid Banner
	Guangxi	Baise City	Debao County
			Jingxi City
		Hechi City	Fengshan County
			Bama Yao Autonomous County
	Chongqing	Chengkou County	Chengkou County
	Sichuan	Pengshui County	Rangtang County
		Aba (Ngawa) Tibetan and Qiang Autonomous Prefecture	Aba County
			Ruoergai County
			Hongyuan County
			Xinlong County
	Guizhou	Qiandongnan Miao and Dong Autonomous Prefecture	Jinping County
			Luodian County
	Yunnan	Chuxiong City	Wuding County
		Nujiang City	Gongshan Derung and Nu Autonomous County
		Deqen Prefecture	Deqin County
	Shaanxi	Hanzhong City	Zhenba County
		Ankang City	Langao County
		Shangluo City Huangnan City	Shangnan County
			Zhashui County
	Qinghai	Guoluo Prefecture	Jianza County
		Chuxiong City	Maqin County
			Banma County
Counties constrained by social development capacity (counties in category III)	Chongqing	Wuxi County	Wuxi County
		Youyang County	Youyang Tujia and Miao Autonomous County
		Pengshui County	Pengshui miao and tujia autonomous county
	Guizhou	Liupanshui City	Shuicheng District
		Zunyi City	Zheng’an County
		Anshun City	Guanling Buyei and Miao Autonomous County
		Bijie City	Zhijin County
			Nayong County
			Weining Yi, Hui and Miao Autonomous County
			Hezhang County
		Tongren City	Yanhe Tujia Autonomous County
			Miao Autonomous County of Songtao
		Qianxinan Prefecture	Wangmo County
	Yunnan	Qujing city	Xuanwei City
		Zhaotong City	Daguan County
			Zhenxiong County
			Yiliang County
		Wenshan Prefecture	Maguan County
	Shaanxi	Ankang City	Ziyang County
	Gansu	Tianshui City	Qin’an County
			Zhangjiachuan Hui Autonomous County
Counties constrained by environmental carrying capacity (counties in category IV)	Inner Mongolia	Xilingol League	Zhengxiangbai Banner
	Guizhou	Zunyi City	Wuchuan Gelao and Miao Autonomous County
		Qiandongnan Prefecture	Qinglong County
	Yunnan	Kunming City	Dongchuan
		Zhaotong City	Yongshan County
		Nujiang City	Lushui City
	Shaanxi	Ankang City	Hanbin District
	Gansu	Tianshui City	Wheat plot area
		Pingliang City	Jingning County
		Gannan Prefecture	Lintan County
Counties constrained by economic agglomeration–Social development capacity (counties in category V)	Guangxi	Liuzhou	Rongshui Miao Autonomous County
			Dong Autonomous County of Sanjiang
		Baise City	Lingyun County
			Tianlin County
		Hezhou City	Zhaoping County
		Hechi City	Donglan County
			Luocheng Mulao Autonomous County
			Huanjiang Maonan Autonomous County
			Du’an Yao Autonomous County
			Dahua Yao Autonomous County
		Laibin City	Xincheng County
		Chongzuo City	Tianhou County
	Sichuan	Ganzi Prefecture	Dege County
		Liangshan Prefecture	Leibo County
	Guizhou	Anshun City	Ziyun Miao and Buyei Autonomous County
		Qianxinan Prefecture	Ceheng County
		Qiandongnan Prefecture	Jianhe County
			Rongjiang County
			Congjiang County
			Sandu Shui Autonomous Condado
	Yunnan	Honghe City	Honghe County
		Wenshan Prefecture	Guangnan County
		Nujiang City	Fugong County
			Lanping Bai and Pumi Autonomous County
		Deqen Prefecture	Weixi Lisu Autonomous County
	Shaanxi	Hanzhong City	Qingyang County
		Shangluo City	Shanyang County
			Zhen’an County
	Gansu	Pingliang City	Zhuanglang County
